# Norovirus genotype distribution in outbreaks of acute gastroenteritis among children and older people: an 8-year study

**DOI:** 10.1186/s12879-016-1999-8

**Published:** 2016-11-07

**Authors:** Makoto Kumazaki, Shuzo Usuku

**Affiliations:** 1 Microbiological Testing and Research Division, Yokohama City Institute of Public Health, Kanagawa, Japan; 2 Yokohama City Institute of Public Health, 2-7-1 Tomiokahigashi, Kanazawa-ku, Yokohama, Kanagawa 236-0051 Japan

**Keywords:** Norovirus, Acute gastroenteritis, Outbreak, Genotype, Epidemiology, Genetic analysis

## Abstract

**Background:**

Noroviruses (NoVs) are the most frequent cause of acute gastroenteritis worldwide among people of all ages and the leading cause of gastrointestinal disease outbreaks in various settings. To clarify the differences in epidemic situations among different settings, we investigated epidemiological trends and the distribution of NoV genotypes in Yokohama, Japan.

**Methods:**

Between September 2007 and August 2015, 746 outbreaks of NoV gastroenteritis were reported in kindergarten/nursery schools (K/Ns), primary schools (PSs), and nursing homes for the aged (NHs). Stool samples were collected for NoV testing, and the NoV gene was amplified and sequenced to determine the genotype.

**Results:**

During the eight seasons, 248 NoV outbreaks occurred in K/Ns, 274 outbreaks in PSs, and 224 outbreaks in NHs. These outbreaks occurred throughout the year, except in August, and the number increased in November and peaked in December. The number of outbreaks that occurred from November to February comprised 76.8 % of all outbreaks. The outbreaks originated in K/Ns or PSs in every season, except for one season. Five genogroup (G)I and nine GII genotypes in K/Ns, six GI and 10 GII genotypes in PSs, and three GI and six GII genotypes in NHs were detected during the eight seasons. GII.4 was the most prevalent genotype in K/Ns and NHs. However, GII.6 was the most prevalent genotype in PSs. The epidemic genotypes in K/Ns and PSs changed by NoV season, although GII.4 was always predominant in NHs. Moreover, the distribution of genotypes was significantly different between epidemic and non-epidemic periods in each facility (*p* < 0.01 for all).

**Conclusions:**

The epidemic situation of NoV outbreaks differs by facility, NoV season, and month. The genotype distribution is likely dependent on the facility and is significantly different between epidemic and non-epidemic periods.

## Background

Noroviruses (NoVs) are single-stranded positive-sense RNA viruses in the family *Caliciviridae*. Based on their capsid gene, NoV strains can be classified into six genogroups (GI–GVI), of which GI, GII, and GIV infect humans [1]. NoV GI contains nine genotypes, and NoV GII contains 22 genotypes [2]. Globally, NoV GII strains are dominant, and GII.4 is reportedly the predominant NoV genotype [3].

NoVs can spread through contaminated food or water or from person to person and are highly infectious [1, 4]. They are the most common pathogenic cause of gastrointestinal disease outbreaks [1]. NoV outbreaks are common in many settings such as health care settings, schools, and food services worldwide [5–8]. Waterborne outbreaks have also been reported [9, 10]. In Yokohama, Japan, NoV outbreaks have occurred in various settings annually, mainly in kindergarten/nursery schools (K/Ns), primary schools (PSs), and nursing homes for the aged (NHs) [11].

To examine the trends of epidemic NoV infectious disease, epidemiological studies have been conducted focused on the transmission route or setting [5, 7, 12–14]. The relationship between outbreak settings and NoV genotypes has been also studied [6, 7]. Among these, the relationship between long-term care facilities or health care settings and GII.4 has often been reported [6, 14]. Additionally, molecular epidemiological studies involving systematic surveillance of all age groups and routes of infection have been performed [15]. However, to our knowledge, longitudinal studies comparing the epidemiological and genetic characteristics of NoV outbreaks in K/Ns, PSs, and NHs have not been reported, especially studies concerning the difference in genotypes detected in these facilities between epidemic and non-epidemic periods using statistical analyses. Moreover, the development of NoV vaccines is currently underway [16, 17]. Thus, understanding the epidemic situation of NoV and the distribution of genotypes will help to determine the utility of future vaccines.

To clarify the difference in epidemic situations among these facilities, we investigated epidemiological trends of NoV outbreaks in K/Ns, PSs, and NHs that occurred in Yokohama, Japan. We also performed a genetic analysis of the strains isolated in these facilities to determine the distribution of NoV genotypes.

## Methods

### Sample collection

Outbreaks of gastroenteritis in Japan are reported to local government public health centers by order of the Ministry of Health, Labour and Welfare. The local government public health centers then conduct field investigations. Between September 2007 and August 2015, 746 outbreaks of NoV gastroenteritis, suspected to be due to foodborne or person-to-person transmission, were reported in K/Ns, PSs, and NHs. 3,500 stool samples were collected for NoV testing by the Health and Social Welfare Bureau, Yokohama, Japan. The number of K/Ns, PSs, and NHs in Yokohama was 1,501, 342, and 941, respectively. The age groups of patients of each facility were: 0–5 years old in K/Ns, 6–12 years old in PSs, and over 60 years old in NHs. A NoV outbreak was defined as two or more cases of acute gastroenteritis within a week occurring in a given setting, and a NoV season was defined as the 12-month period from September through August of each year.

### Detection of the NoV gene using real-time RT-PCR

A 10 % stool suspension was prepared by mixing each stool sample with phosphate-buffered saline, followed by centrifugation at 10,000 × *g* for 10 min at 4 °C. Viral RNA was extracted from the supernatants using the RNeasy Mini Kit (Qiagen, Hilden, Germany) according to the manufacturer’s instructions. Real-time RT-PCR detection of NoV was performed using a Smart-Cycler II (Cepheid, Sunnyvale, CA, USA) using the QuantiTect Probe RT-PCR Kit (Qiagen) with separate reactions for NoV GI and II. The primers and probes used to detect these viruses have been described elsewhere [18, 19].

### RT-PCR for NoV genotyping

One positive specimen selected randomly from each patient in each NoV outbreak was subjected to gene amplification of region C (the 5′ end of open reading frame 2) to determine the genotype. RT-PCR was performed using the TaKaRa One Step RNA PCR Kit (Takara Bio Inc., Shiga, Japan). The primers used for PCR have been described elsewhere [20, 21].

### Sequencing and NoV genotyping

The nucleotide sequences of the purified PCR products (QIAquick PCR Purification Kit, Qiagen) were determined using the BigDye Terminator Cycle Sequencing Kit (Applied Biosystems, Foster City, CA, USA) and a Genetic Analyzer 3130 (Applied Biosystems) according to the manufacturer’s instructions. The obtained data were used to determine the genotype using the web-based Norovirus Genotyping Tool Version 1.0 software program [22].

### Statistical analysis

Statistical analysis was performed using IBM SPSS Statistics Version 22 (IBM, Armonk, NY, USA). Pearson’s correlation analysis or Fisher’s exact test was used to determine significant differences between genotypes in epidemic and non-epidemic periods. *P* values < 0.01 were considered to be statistically significant.

## Results

### NoV outbreaks in K/Ns, PSs, and NHs

During eight consecutive 12-month periods starting in September 2007, 248 NoV outbreaks occurred in K/Ns, 274 outbreaks in PSs, and 224 outbreaks in NHs. The majority of these outbreaks were suspected to be due to person-to-person transmission by epidemiological investigation. A summary of the NoV outbreaks in each season is listed in Table 1. Outbreaks primarily occurred in K/Ns in the 2010–2011, 2013–2014, and 2014–2015 seasons; in PSs, in the 2007–2008, 2008–2009, and 2009–2010 seasons; and in NHs, in the 2011–2012 and 2012–2013 seasons.

**Table 1:** Summary of NoV outbreaks reported in Yokohama, Japan from September 2007 to August 2015

	K/Ns			PSs			NHs		
	0–5 years old			6–12 years old			>60 years old		
Season	Outbreaks	Tested samples	Positive samples	Outbreaks	Tested samples	Positive samples	Outbreaks	Tested samples	Positive samples
2007–2008	10	50	36	33	267	153	28	200	90
2008–2009	16	74	60	41	181	133	21	148	73
2009–2010	10	58	36	43	173	138	17	57	53
2010–2011	51	218	145	47	151	129	21	96	66
2011–2012	28	112	85	21	79	63	34	139	103
2012–2013	39	195	132	24	74	67	58	328	204
2013–2014	57	207	153	40	150	120	25	116	85
2014–2015	37	180	133	25	141	87	20	106	69

### Monthly distribution of NoV outbreaks

**Fig. 1:**
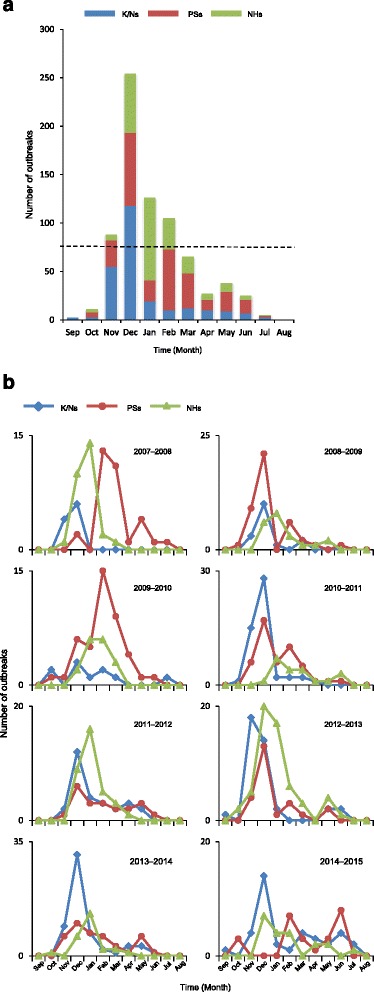
NoV outbreaks in K/Ns, PSs, and NHs in Yokohama, Japan, 2007–2015. **a** Cumulative number of NoV outbreaks. The dashed line indicates 10 % (N = 74.6) of all outbreaks; **b** Monthly distribution of NoV outbreaks

The monthly cumulative number of outbreaks in K/Ns, PSs, and NHs is shown in Fig. 1a. NoV outbreaks occurred throughout the year, except in August, and the number increased in November and peaked in December. From November to February (the winter season), the number of monthly outbreaks accounted for over 10 % of the overall total (Fig. 1a). The number of outbreaks during these 4 months comprised 76.8 % of the overall total. When we examined the outbreaks in the three facilities, outbreaks in K/Ns mainly occurred in November–December (Fig. 1a). The number of outbreaks in these 2 months corresponded to 69.8 % of all outbreaks in K/Ns. After January, the number of outbreaks rapidly declined. Outbreaks in PSs mainly occurred in December and February (Fig. 1a) and corresponded to 50.4 % of all outbreaks in PSs. Outbreaks in NHs mainly occurred in December–January (Fig. 1a) and corresponded to 65.2 % of all outbreaks in NHs. After February, the number of outbreaks gradually declined.

The monthly outbreak distribution of each season is shown in Fig. 1b. In K/Ns, out of eight seasons, six seasons peaked in December. The 2012–2013 season peaked in November, whereas no clear peak was observed in the 2009–2010 season. The monthly distribution of outbreaks in PSs differed each season. Out of eight seasons, five seasons peaked in December and two seasons peaked in February. However, in the 2014–2015 season, the number of outbreaks peaked in June, which is early summer in Japan. Of note, outbreaks originated in K/Ns or PSs in each season, except for the 2013–2014 season (Fig. 1b). Conversely, in NHs, out of eight seasons, five seasons peaked in January. The 2009–2010 season peaked during January–February, and the 2012–2013 and 2014–2015 seasons peaked in December.

### NoV genotypes detected in K/Ns, PSs, and NHs

In K/Ns, 232 (93.5 %) outbreaks were caused by GII, 14 (5.7 %) outbreaks by GI, and two (0.8 %) outbreaks by a mixture of GI and GII. Similarly, 198 (72.3 %) outbreaks were caused by GII, 65 (23.7 %) outbreaks by GI, and 11 (4.0 %) outbreaks by a mixture of GI and GII in PSs. In NHs, 221 (98.7 %) outbreaks were caused by GII, three (1.3 %) outbreaks were caused by GI, and none was caused by a mixture of GI and GII. Five GI genotypes (GI.2, GI.3, GI.4, GI.6, and GI.7) and nine GII genotypes (GII.2, GII.3, GII.4, GII.5, GII.6, GII.7, GII.12, GII.14, and GII.17) in K/Ns, six GI (GI.2, GI.3, GI.4, GI.5, GI.6, and GI.7) and 10 GII (GII.2, GII.3, GII.4, GII.6, GII.7, GII.12, GII.13, GII.14, GII.15, and GII.17) genotypes in PSs, and three GI (GI.3, GI.4, and GI.6) and six GII (GII.2, GII.3, GII.4, GII.6, GII.12, and GII.17) genotypes in NHs were detected during the eight seasons. GII.4 was the most prevalent genotype in K/Ns and NHs and corresponded to 40.7 % and 93.8 % of the total, respectively, in each facility. Conversely, GII.3 (23.4 %), GII.6 (12.9 %), GII.14 (6.9 %), and GII.2 (6.0 %) were prevalent genotypes followed by GII.4 in K/Ns. The eight genotypes, except GII.4, detected in NHs corresponded to less than 2 % of the total. However, in PSs, GII.6 was the most prevalent genotype and corresponded to 19.7 % of the total. Of note, GII.2 (17.9 %), GII.14 (10.2 %), GII.3 (9.5 %), and GI.4 (8.8 %) were more frequently detected than GII.4 (8.4 %) in PSs.

### Trends of NoV genotypes

**Fig. 2:**
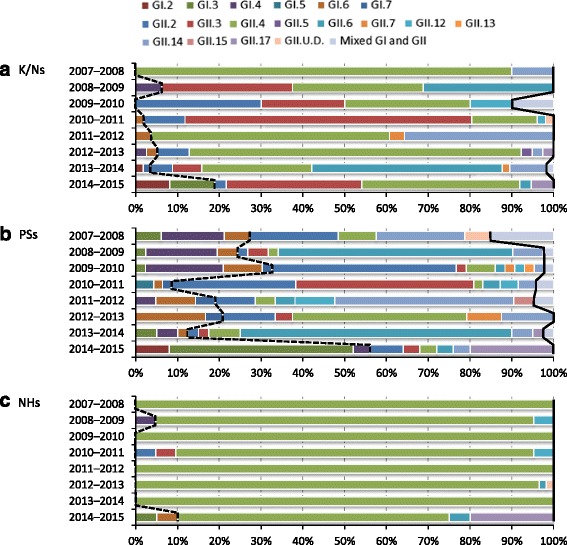
Distribution of NoV capsid genotypes in (**a**) K/Ns, (**b**) PSs, and (**c**) NHs by season. The bars on the left side of the dashed line indicate GI genotypes, the bars between the dashed line and the solid line indicate GII genotypes, and the bars on the right side of the solid line indicate the mixture of GI and GII genotypes. GII.U.D., undetermined genotypes in GII

We also analyzed the genotypes detected in each season (Fig. 2a–c). Two to nine genotypes were detected in K/Ns, 7–13 genotypes in PSs, and 1–5 genotypes in NHs for each season.

In detail, GII was predominant in all 8 seasons, and GII.4 was most prevalent in four of the eight seasons in K/Ns (Fig. 2a). GII.4 accounted for 15.7–90.0 % of outbreaks in each season. GII.3, GII.4, and GII.6 were equally dominant in the 2008–2009 season (31.3 % each), and GII.2 and GII.4 were equally dominant in the 2009–2010 season (30.0 % each). GII.3 was the most prevalent in the 2010–2011 season (68.6 %), and GII.6 was predominant in the 2013–2014 season (45.6 %). GI accounted for 0–18.9 % of outbreaks in each season (Fig. 2a).

GII was predominant in seven of the eight seasons, and alterations in the dominant genotype in each season were observed in PSs (Fig. 2b). GII.6 was the most prevalent genotype in the 2008–2009 and 2013–2014 seasons (56.1 % and 65.0 %, respectively), whereas GII.2 in the 2009–2010 season (44.2 %), GII.3 in the 2010–2011 season (42.6 %), and GII.14 in the 2011–2012 season (42.9 %) were most prevalent. GII.2 and GII.14 were equally dominant in the 2007–2008 season (21.2 % each). Of note, GII.4 was the most prevalent genotype only in the 2012–2013 season (41.7 %), which is when a new GII.4 variant (Sydney 2012) spread worldwide. GI accounted for 8.5–56.0 % of outbreaks in each season, and GI was predominant only in the 2014–2015 season. In this season, GI.3 was the most prevalent genotype (44.0 %) among all genotypes (Fig. 2b).

Although GII.4 was predominant in NHs, its proportion changed in each season (Fig. 2c). GII.4 comprised 85.7–100 % of the total between the 2007–2008 and 2013–2014 seasons. However, GII.4 accounted for only 65.0 % of the total in the 2014–2015 season, and GII.17 accounted for 20.0 %. The GII.17 strains detected in this season were phylogenetically similar to strains that have been reported as a new variant, GII.17 Kawasaki (Kawasaki308; LC037415), and these strains primarily circulated in Asia in 2014 and 2015 [23–25].

### Analysis of genotypes detected in epidemic and non-epidemic periods

**Fig. 3:**
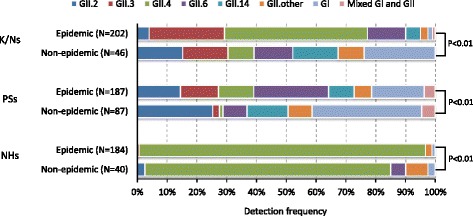
Comparison of the distribution of NoV capsid genotypes in epidemic and non-epidemic periods. Epidemic indicates the epidemic period (November–February), and Non-epidemic indicates the non-epidemic period (September, October, and March–August). GII.U.D., undetermined genotypes in GII

In this study, we tentatively defined an epidemic period in Yokohama from November to February in each NoV season because the number of monthly outbreaks accounted for over 10 % of the overall total (Fig. 1a). We conducted a correlation analysis to compare the genotypes detected in the epidemic period (November to February) and the non-epidemic period (September, October, and March–August). As shown in Fig. 3, a significant difference in the proportion of genotypes was observed between epidemic and non-epidemic periods in each facility (*p* < 0.01 for all).

In PSs, GII.3, GII.4, and GII.6 were more frequently detected in the epidemic period (12.8, 11.8, and 25.1 %, respectively) than the non-epidemic period (2.3, 1.1, and 8.0 %, respectively). Conversely, GII.2, GII.14, and GI genotypes were more frequently detected in the non-epidemic period (25.3, 13.8, and 36.8 %, respectively) than the epidemic period (14.4, 8.6, and 17.6 %, respectively). In K/Ns, GII.3 and GII.4 were more frequently detected in the epidemic period (25.2 and 48.0 %, respectively) than the non-epidemic period (15.2 and 8.7 %, respectively). Similar to PSs, GII.2, GII.14, and GI genotypes were more frequently detected in the non-epidemic period (15.2, 15.2, and 23.9 %, respectively) than the epidemic period (4.0, 5.0, and 1.5 %, respectively). In NHs, GII.4 was more frequently detected in the epidemic period (96.2 %) than the non-epidemic period (82.5 %). Common to all three facilities, GII.4 was more frequently detected in the epidemic period, and GII.other (all GII genotypes except GII.2, GII.3, GII.4, GII.6, and GII.14) and GI genotypes were more frequently detected in the non-epidemic period (Fig. 3).

## Discussion

In this study, we focused on three facilities, K/N, PS, and NH, and investigated the epidemic situation of NoV outbreaks and distribution of genotypes between 2007 and 2015 in Yokohama, Japan. The number of outbreaks in these three facilities accounted for approximately 70–80 % of the total NoV outbreaks reported in Yokohama, although the magnitude of outbreaks differed in each facility annually [26]. 76.8 % of the outbreaks in these three facilities occurred in November–February, and the highest prevalence of NoV outbreaks was observed in the winter period. This finding is consistent with other reports which confirmed the clear winter seasonality of NoV outbreaks [27]. It is considered that a high number of NoV cases in winter relate with cold temperatures and dry conditions in Japan [28].

During September–November, which is the beginning of the NoV season, the number of outbreaks was highest in K/Ns, followed by PSs, and NHs. Moreover, outbreaks originated in K/Ns or PSs in each season, except for one season. These outbreaks among infants and children likely led to outbreaks among older people. Various NoV genotypes are present in the environment such as rivers, sewage, and food throughout the year, although the prevalences can fluctuate [29–31]. NoVs can also be transmitted by asymptomatic people [32]. Younger people, such as infants and children, may be more susceptible to many NoV genotypes because they have not acquired sufficient immunity. Thus, it is likely that NoV outbreaks initially spread among young people. Additionally, a sharp increase in outbreaks was observed in K/Ns compared with the other two facilities. In general, the norovirus load decreases with increasing age, especially in young children aged <5 years, although the median norovirus load of any genotype is high [33]. A higher norovirus load or the environment in K/Ns might affect the rapid spread of the infection. Furthermore, especially in K/Ns and NHs, the outbreaks in the 2012–2013 season occurred earlier than in the other seasons. This might have been related to the emergence of a new GII.4 variant (Sydney 2012) [26].

During this study period, 14 genotypes in K/Ns, 16 genotypes in PSs, and nine genotypes in NHs were detected during NoV outbreaks. Sakon et al. also reported that the number of genotypes detected in children (0–14 years old) was greater than that in older people (>65 years old) [15]. The proportion of detected genotypes differed between K/Ns and PSs, although GII.2, GII.3, GII.4, GII.6, and GII.14 were common epidemic genotypes in the two facilities. GII.6 was the most prevalent genotype in PSs, whereas GII.4 was the most prevalent genotype in K/Ns. Additionally, the proportion of GI in PSs (23.7 %) was higher than that in K/Ns (5.7 %). The infants attending K/Ns are in closer contact with adults than school children, and GII.4 is the dominant genotype in adult populations [15]. Moreover, several studies confirmed that GI genotypes are present in the environment, although GI genotypes are detected less frequently in clinical samples than GII genotypes [6, 26, 29, 30, 34]. It is thus likely that school children come in contact with various genotypes, including GI genotypes, because school children have a wider sphere of activity than infants. Indeed, another study has also reported that GI strains were found relatively more often in schools [12]. Conversely, the most prevalent genotype in NHs was GII.4, which is consistent with other reports on older people [6, 14, 15, 35]. In NHs, the infectious route of GII.4 likely occurs through person-to-person transmission through helpers or visitors. However, it is unclear why GII.4 is predominant in adults and older people [14, 15]. Moreover, the genotypes in community-acquired NoV infections are more heterogeneous than in nosocomial infection [35]. Thus, the genotype detected in NHs was homogeneous likely because people in NHs have limited mobility and live in a confined space.

The epidemic genotypes in K/Ns and PSs changed by NoV season, whereas GII.4 remained the predominant genotype in NHs. GII.2, GII.3, GII.6, and GII.14 were detected throughout Yokohama during this study period [26], and these four genotypes are mainly associated with sporadic infections in children in some Asian countries, including Japan [8, 15, 36, 37]. Genotype-specific herd immunity in infants and young children can last for years and likely influences the endemic NoV genotype(s) in the next season [15]. Moreover, GII.4 was the most prevalent genotype in PSs only in the 2012–2013 season. The GII.4 Sydney 2012 variant spread worldwide during this season [6, 26, 38], and it is likely that the emergence of this variant influenced the epidemic genotypes not only in K/Ns and NHs, but also in PSs. Thus, care is needed when new variants emerge because the infection has a tendency to spread. Conversely, GII.17 rapidly increased in the 2014–2015 season in NHs and PSs, although GII.17 was rarely detected in Yokohama previously [26]. Although GII.17 only accounted for two outbreaks (5.4 %) in K/Ns in the 2014–2015 season, GII.17 outbreaks accounted for 20 % of the total in this season in PSs and NHs. The median age of GII.17 cases was significantly older than that of GII.4 cases, with a wide age distribution of GII.17 infections in Hong Kong [24]. Further data collection and analysis are needed to understand the potential age specificity of patients infected with GII.17.

Our study also revealed that the proportions of genotypes detected in each facility were significantly different between epidemic and non-epidemic periods. GII.4 outbreaks increased in winter [3], which is consistent with our result that GII.4 was significantly more frequently detected in the epidemic period (winter in Yokohama) in K/Ns, PSs, and NHs. Moreover, it has been reported that other GII genotypes, except GII.4, exhibited a weaker seasonal pattern [12], whereas non-GII.4 outbreaks caused by person-to-person transmission occurred significantly less in the fall and winter seasons and significantly more in the spring-summer season [6]. However, in our study, the distribution of GII.2, GII.3, GII.6, and GII.14 significantly differed between epidemic and non-epidemic periods. Among these genotypes, GII.3 occurred significantly more frequently in the epidemic period in K/Ns and PSs. GII.6 also occurred significantly more frequently in the epidemic period in PSs. Thus, some genotypes, except GII.4, exhibited seasonality. Further investigations are necessary to determine precisely the correlations between genotypes and outbreak setting and season.

## Conclusions

We revealed that the epidemic situation of NoV differed by facility, NoV season, and month even in the same geographical area. The distribution of genotype is likely dependent upon the facility, especially the patient age. Moreover, our study revealed that the proportions of genotypes detected in each facility were significantly different between epidemic and non-epidemic periods. Understanding the epidemic situation of NoV and the distribution of genotypes will help to determine the utility of future vaccines.

## Abbreviations

G: Genogroups
K/Ns: Kindergarten/nursery schools
NHs: Nursing homes for the aged
NoVs: Noroviruses
PSs: Primary schools
